# Common occurrence of atrophic gastritis in an ageing non-hospitalised population: an autopsy study

**DOI:** 10.1093/ageing/afaf047

**Published:** 2025-03-03

**Authors:** Pekka Karhunen, Sari Tuomisto, Sirkka Goebeler, Mika Martiskainen, Eloise Kok

**Affiliations:** Faculty of Medicine and Health Technology, Tampere University, Tampere, Finland; Fimlab Laboratories Ltd, Tampere, Pirkanmaa, Finland; Faculty of Medicine and Health Technology, Tampere University, Tampere, Finland; Fimlab Laboratories Ltd, Tampere, Pirkanmaa, Finland; Finnish Institute for Health and Welfare, Helsinki, Uusimaa, Finland; Faculty of Medicine and Health Technology, Tampere University, Tampere, Pirkanmaa, Finland; Finnish Institute for Health and Welfare, Helsinki, Uusimaa, Finland; Department of Medicine and Health Technology, Tampere University, Tampere, Finland; Faculty of Medicine and Health Technology, Tampere University, Tampere, Pirkanmaa, Finland; Faculty of Medicine, Department of Pathology, University of Helsinki, Helsinki, Uusimaa, Finland

**Keywords:** gastritis, atrophic, chronic, *H. pylori*, older people

## Abstract

**Background:**

Atrophic gastritis—the end stage of chronic gastritis—is an asymptomatic disease due to *Helicobacter pylori* infection causing decreased vitamin B12 and folate absorption, which may lead to severe haematological and neuropsychological disorders including Alzheimer’s disease. The diagnosis requires endoscopy and biopsies from symptomatic patients, explaining why its true prevalence in the population is not well-known.

**Objective:**

We aimed to evaluate the prevalence of various stages of chronic gastritis in an autopsy series most closely representing the general population.

**Subjects and Methods:**

Gastric mucosa samples were collected prospectively from out-of-hospital deaths included in the Tampere Sudden Death Study (*n* = 70, mean age 63, age range 22–91 years). Antrum and corpus samples were stained with a *H. pylori* antibody and staged histopathologically.

**Results:**

Chronic gastritis with or without atrophic changes was detected in 40% of the cases. The proportion of healthy mucosa decreased age-dependently from 71.4% among individuals aged <50 years to 43.5% among the oldest individuals (>70 years), and that of chronic non-atrophic gastritis from 21.4% to 8.7%. In contrast, the prevalence of atrophic gastritis was 27.1% and increased in the age groups from 7.1% to 47.8% (*P* = .019) among the oldest individuals, showing a strong association (*P* < .0001) with *H. pylori* immunopositivity.

**Conclusions:**

Atrophic gastritis is a common feature of the ageing stomach, which is observed in every second individual aged 70+ years, showing a strong association with *H. pylori* immunopositivity. Atrophic gastritis may be a more common risk factor in old age for diseases associated with low serum B12 and folate levels than has been previously known.

## Key Points

- Atrophic gastritis is a common feature of the ageing stomach being seen in every second individual aged 70+ years.
- Atrophic gastritis may be a more common than previously known risk factor at old age for diseases associated with low serum B12.
- Atrophic gastritis shows strong association with *Helicobacter pylori* infection

## Introduction

Chronic gastritis is a common disease with potentially serious consequences. More than half of the world’s population may be affected by the disease to some extent, although its prevalence has declined in Western countries in recent years, along with a decrease in the prevalence of *H. pylori* infections—the main cause of chronic gastritis [1, 2]. Addressing chronic gastritis is, nonetheless, very important, as the end stage of the disease—atrophic gastritis—is a key risk factor in gastric cancer [3–5], but also because it decreases the absorption of cobalamin (vitamin B12) [6, 7] and folate [8], leading to an increased risk of severe haematological disorders such as pernicious anaemia, and a broad spectrum of neurological, cognitive and psychiatric manifestations [6, 7]. Low plasma levels of vitamin B12 and folic acid increase homocysteine levels, which have been linked with cognitive decline and with an increased risk of dementia [6, 7, 9, 10] as well as with increased risk of ischaemic heart disease [11]. The real risk that atrophic gastritis poses for these diseases is difficult to estimate because the prevalence of atrophic gastritis in old age in an asymptomatic population has not been ascertained.

According to the guidelines by the British Society of Gastroenterology and the American Gastroenterological Association (AGA) Institute, atrophic gastritis is defined as the process of chronic inflammation mainly caused by *H. pylori* infection or autoimmunity leading to the loss of appropriate glands and their replacement by fibrous tissue or intestinal-type glands which are inappropriate for the location, i.e. metaplasia [12–14]. A diffuse type of atrophic gastritis limited to oxyntic (fundic) mucosa can also be autoimmune in origin, although it seems that *H. pylori* may also play an important role as a microbial trigger in these cases [15–17].

*Helicobacter pylori* is usually acquired during early childhood [18, 19]. Since the infection usually does not disappear spontaneously and primary infection in adults are very rare, the prevalence of the infection in each birth cohort reflects the prevalence of childhood infections, which explains why the prevalence is highest among the older individuals [2, 20]. The declining prevalence of *H. pylori* infection has led to an increased number of *H. pylori* negative gastritis diagnoses worldwide [21, 22]. Recently, a detailed analysis of the gastric microbiota in chronic gastritis and gastric carcinoma using 16S rRNA gene profiling and next-generation sequencing detected an enrichment of other bacterial genera, mostly intestinal commensals, along with *H. pylori* [23].

*Helicobacter pylori* infection usually induces acute gastritis with an active inflammation that lasts a few weeks and subsequent chronic inflammation which may become lifelong [1, 24]. Not all patients with *H. pylori* infections develop chronic gastritis. It is estimated that 20% of acute cases of *H. pylori* gastritis are self-limiting, most likely due to a combination of bacterial strain diversity, host genetics and environmental factors [1, 25, 26]. *Helicobacter pylori* infection tends to appear first in the antrum, developing antrum-predominant chronic gastritis, then spread to the corpus, leading to pan-gastritis. During the acute phase, a histological evaluation of gastric mucosa shows an infiltration of neutrophils. When the infection becomes persistent, neutrophils and other polymorphonuclear leukocytes are gradually replaced by mononuclear inflammatory cells. This stage is referred to as chronic gastritis. As the infection progresses, mononuclear cells form organised lymphoid follicles and the inflammation usually becomes less intense [24, 27]. Atrophic gastritis represents the end stage of this inflammatory sequence, with the process from chronic gastritis to chronic gastritis with atrophic changes (atrophic gastritis) advancing gradually over decades, usually while the patient remains asymptomatic [19, 28–30]. Heartburn and regurgitation are present in ~24% and 12% of the patients, respectively [30].

According to the guidelines, the diagnosis of atrophic gastritis should be confirmed by means of histopathology [13, 14] The actual prevalence of chronic gastritis and chronic atrophic gastritis in a community-dwelling population remains unclear; however, as the disease is often asymptomatic [19, 29] and the golden standard for diagnosis is a histological evaluation of biopsies from invasive endoscopies. In a meta-analysis of 14 studies covering 18,510 cases, the prevalence of atrophic gastritis in the general population was suggested to be ~16% [31]. There are no data on the prevalence of less advanced forms of chronic gastritis in the general population. However, in a cohort of Finnish outpatients who underwent gastroscopy due to gastrointestinal symptoms, the prevalence of chronic gastritis amounted to as high as 80% among those aged 70+ years and that of atrophic gastritis to 40% [2]. The only non-invasive way to assess the prevalence of atrophic gastritis at a population level is to apply serological assays whose results have not shown good concordance with biopsy studies, generally yielding much lower figures of prevalence [31]. The prevalence of pepsinogen I-defined functional atrophic corpus gastritis was only 5.7% in a study comprising a stratified sample of healthy adults aged 35–64 years from the populations of two small communities in Northern Sweden in 2009 [32]. Among the oldest age group of 55–64 years, the prevalence was 8.3%. Similarly, based on serum pepsinogen I and II assays, the prevalence of atrophic gastritis among New Zealanders was 6.7% [33]. In a systematic meta-analysis review [34], the combination of pepsinogen, gastrin-17 and anti-*H. pylori* antibodies in serological assays (panel test) seemed to be a reliable non-invasive tool for the diagnosis and exclusion of advanced atrophic gastritis, but it does not help in detecting the preceding stages of the disease. As healthy patients cannot ethically be biopsied, autopsy samples may provide a solution, but to the best of the authors’ knowledge, no previous gastritis studies have utilised autopsy series in which the victims had died of reasons unrelated to chronic gastritis.

The purpose of this study was to determine the prevalence and age-dependence of the spectrum of chronic gastritis, and especially that of atrophic gastritis and *H. pylori* infection in an autopsy series most closely representing the general population.

## Subjects and methods

Gastric mucosa samples, one each from the corpus and antrum, were taken from a subset of 74 individuals included in the Tampere Sudden Death Study (TSDS) in 2014–2015 who were subjected to a medicolegal autopsy due to sudden unexpected out-of-hospital death. The average age was 63 years (range 22–91 years), and 79% of the deceased were males (M/F = 59/15). Of the cases, 44 (59.5%) had died of cardiovascular diseases, 11 (14.8%) of other diseases, including two (2.9%) cases (both female, aged 57 and 63 years) who had died of a sudden gastrointestinal haemorrhage due to Mallory–Weiss syndrome. The remaining 19 (25.7%) cases in the subset were non-natural deaths including accidents or suicides. Post-mortem interval data were available for 68 (97.4%) individuals and varied from 1 to 11 days (median, 3 days). Samples were fixed in buffered formaldehyde for at least 24 hours and paraffin-embedded in Tissue-Tek boxes. 5 μm sections were cut and stained with an anti-*H. pylori* antibody immunostain (Novocastra mouse monoclonal antibody from Leica Biosystems UK, 1:100 dilution) and applied using a Labvision LV-1 autostainer. Slides were digitalised with an Operio Scan Scope XT and viewed with the computer programmes ImageJ 1.45 s and JVSview 1.2. This study was approved by the Regional Ethical Committee of Tampere University Hospital (R16199) and the national ethics committee Valvira.

Four cases (5.4%) were excluded from the study due to misrepresentative tissue regions caused by twisting of the thin mucosa samples during fixation and embedding. The final study series thus comprised 70 cases with suitable samples for scoring. The 2 × 2 cm samples showed typical postmortem changes in the mucosa, but this did not significantly affect the scoring as we evaluated the mucosa compartment closest to the muscularis mucosae, which is thought to be the key area for signs of atrophy [35]. The scoring was based on the presence or absence of inflammatory cells and lymphoid follicles, and in the atrophic stage the loss of appropriate glands and their replacement by fibrous tissue or intestinal-type glands (metaplasia). Acute gastritis was defined as a presence of neutrophils and chronic inflammation as an increased number of mononuclear inflammatory cells. Both inflammation and atrophy were classified using the updated Sydney system [27] as absent, mild, moderate or marked. Metaplasia was classified as absent, mild, moderate or marked. Chronic gastritis was assigned when mononuclear inflammation was present with or without atrophic changes. Atrophic gastritis was scored as mild, moderate or marked based on the combination of antrum and corpus scores (see Table 1).

**Table 1:** Characteristics in the Tampere sudden death study (TSDS) cohort (*N* = 70)

	*N*	%
Male	56	80
Female	14	20.0
*H. pylori* positive	20	28.6
Healthy mucosa	42	60.0
Chronic non-atrophic or atrophic gastritis	28	40.0
Chronic non-atrophic gastritis	9	12.9
Chronic atrophic gastritis	19	27.1
Mild atrophy	11	15.7
Moderate atrophy	5	7.1
Severe atrophy	3	4.3

For statistical analyses, the Kruskal–Wallis test, Chi-squared test, *t*-test, Wilcoxon Signed-Rank Test and Spearman’s correlation were used with IBM SPSS statistical software, version 24.

## Results

**Figure 1:**
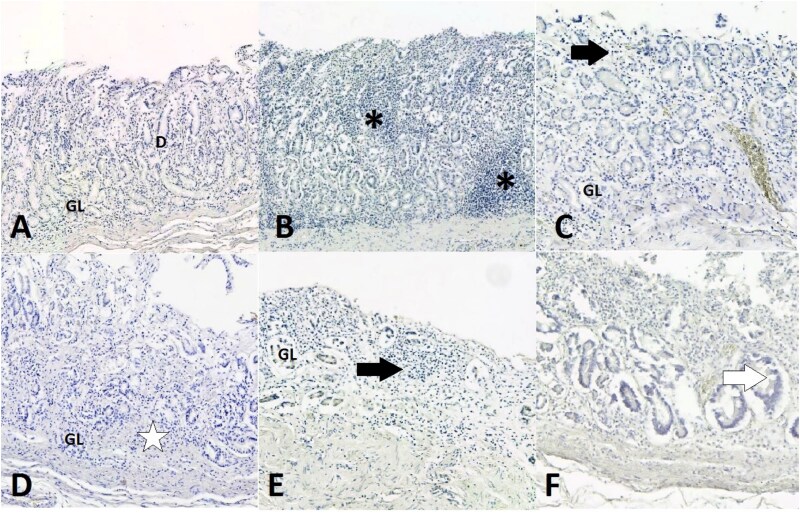
Antrum mucosa (15× magnification) of the TSDS autopsy cases. (A) Healthy mucosa: abundant glands (GL) and no inflammation, with ducts of gastric glands (D) visible. (B) Chronic non-atrophic gastritis: moderate mononuclear inflammation with lymphoid aggregates (asterisks), no neutrophils present and no atrophy. (C) Gastritis with mild atrophy: mild diffuse mononuclear inflammation, such as lymphocytes (black arrow), and mild atrophy where less than 30% of glands (GL) have been replaced by fibrous tissue. (D) Gastritis with moderate atrophy: mild mononuclear inflammation and 30%–60% of glands replaced by fibrous tissue (white star). (E) Gastritis with marked atrophy: mild inflammation and more than 60% of glands (GL) replaced by fibrous tissue. (F) Metaplasia: the architecture of normal gastric mucosa is gone, and glands have been replaced by intestinal-type glands and goblet cells are present (white arrow).

Of the 70 available cases, 56 (80%) were male and 14 (20%) females. The individuals were aged from 22 to 91 years (mean 63 years), with no statistically significant difference in age observed between males and females (*P* = .188). Overall, 42 (60.0%) had normal mucosa and 28 (40.0%) had chronic gastritis with or without atrophic changes. None had acute gastritis. Of the 28 cases with chronic gastritis, 19 (67.9%) had atrophic changes, and 9 (32.1%) had chronic inflammation without atrophy. Of the 19 cases with atrophic gastritis, 11 (57.9%) had mild, 5 (26.3%) moderate and 3 (15.8%) marked atrophy (Figure 1). Metaplastic changes were observed in 5 (26.3%) of the atrophic gastritis cases. Of the cases with mild atrophy, 4 (36.4%) were mainly observed in antrum, 2 (18.2%) in the corpus and 5 (45.4%) in both areas. As expected, 5 (62.5%) of the eight cases with moderate or severe atrophy were detected in both antrum and corpus samples. No statistically significant difference in the prevalence of gastritis was observed between women and men. Chronic gastritis with or without atrophy tended (OR 0.53; 95% CI: 0.15–1.91) to be more common among men (42.9%) compared to women (28.6%), whereas the frequencies of atrophic gastritis were similar for both sexes (26.8% vs. 28.6%, respectively; OR 1.09; 95% CI: 0.30–4.02).

**Figure 2:**
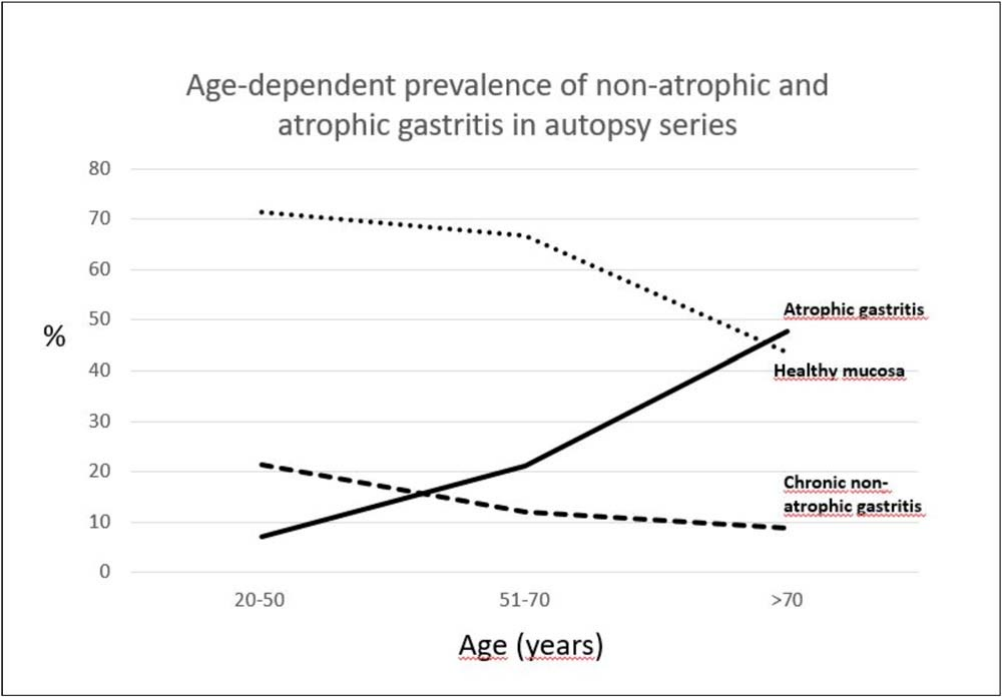
The prevalence of atrophic gastritis increases with age.

The overall prevalence of atrophic gastritis in this cohort was 27.1%. The prevalence of atrophic gastritis increased with age (Figure 2). The prevalence of atrophic gastritis in the age group of 20–50-year-olds was 7.1%, in those aged 51–70 years 21.2% and in the group aged 71–91 years 47.8% (*P* = .019).

**Figure 3:**
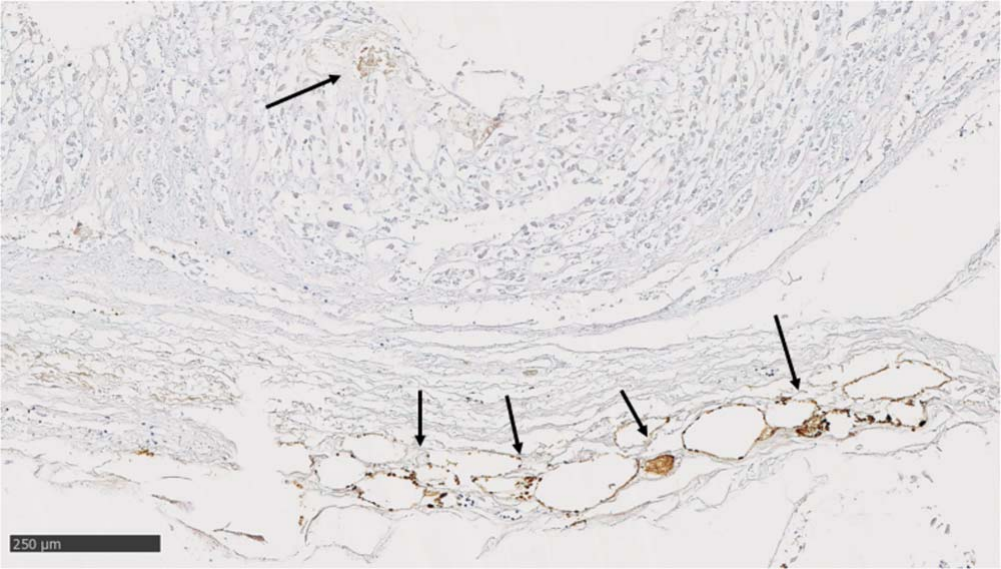
Atrophic mucosa showing *Helicobacter pylori* colonisation (arrows).

*Helicobacter pylori* was detected in 20 (28.6%) cases (Figure 3). Of the 28 cases with chronic gastritis with or without atrophy, 18 (64.3%) were positive for *H. pylori*. The prevalence of *H. pylori* was 4.8% in the healthy mucosa subgroup, 44.4% in those with chronic non-atrophic gastritis and 73.5% in those with chronic atrophic gastritis (*P* < .001). The prevalence of *H. pylori* was 14.3% in those aged 20–50 years, 27.3% in those aged 51–70 years and 39.1% in the age group of 71–91-year-olds, but no statistically significant difference was found between the age groups (*P* = .261. No statistically significant difference in *H. pylori* positivity was observed between women and men (OR: 1.00; 95% CI: 0.274–3.656, *P* = 1.00).

Of the cases, 14 (20.0%) showed a colonisation of bacteria other than *H. pylori*, but no statistically significant correlation was detected between bacterial colonisation and the postmortem interval (*P* = .866).

## Discussion

In this study cohort, atrophic gastritis was found to be relatively common, with the prevalence and severity increasing with age. Atrophic changes were typically mild (57.9% of all atrophic cases) and multifocal. The changes were particularly common among the oldest individuals, with half of the autopsy cases older than 70 years having atrophic changes, although these changes were usually only mild or moderate. The prevalence of atrophic gastritis was greater than the prevalence of non-atrophic chronic gastritis. Only corpus atrophy leads to functional changes such as impaired gastric acid and intrinsic factor secretion or iron and cobalamin deficiency. In all our atrophic gastritis cases, the corpus mucosa was more or less affected in 36.8%.

The present study showed that *H. pylori* bacteria were also detected in some cases with virtually normal mucosa, suggesting that *H. pylori* colonisation does not necessarily lead to inflammation. Interestingly, 26.3% of the cases with atrophic gastritis and 55.5% of those with chronic gastritis without atrophy were *H. pylori* negative. Some may be false negatives as a result of a failure to detect the bacteria, perhaps due to the fact that *H. pylori* bacterial numbers decrease—sometimes beyond detection—as the atrophy advances [36], but it is unlikely that all of the negative cases were false negatives. Autoimmune gastritis could also explain the number of *H. pylori* negative gastritis cases, but there was only one sample (1.4%) with the appearance of autoimmune gastritis.

Few gastritis studies have been conducted on non-symptomatic individuals. A study [37] conducted on 4256 volunteers (mean age 56 years) in Finland found the seroprevalence of *H. pylori* to be 19%, and the prevalence of moderate or severe atrophic corpus gastritis 3.5% overall, and, among those older than 70 years, 8.0%. In that study, a diagnosis of advanced atrophic corpus gastritis was made from blood samples with the GastroView® biomarker (which has 82% sensitivity) and limited to corpus atrophy. Our autopsy series representing a community-dwelling normal population (mean age 63 years) showed somewhat similar results for the prevalence of *H. pylori* (28.6%). In contrast, the prevalence of all forms of atrophic gastritis in the oldest age group of 70 + years was 47.8%—a much higher figure than the previous study based on the use of biomarkers. Another study [38] conducted in 1994 in a nearby region of Finland found a 33.0% seroprevalence of *H. pylori* among randomly selected people born in 1940–1949. In our study, most individuals were born in the 1940’s and 1950’s, therefore our 28.6% prevalence of *H. pylori* concurs well with this previous study. Non-invasive studies based on the measurement of serum biomarkers can thus be used to screen for gastric atrophy, but cut-off values are usually set to differentiate advanced atrophic gastritis from healthy mucosa, thereby offering little information on cases with chronic non-atrophic gastritis or gastritis with mild atrophic changes, which may also have clinical significance. An endoscopic examination can reveal signs of inflammation, but there is poor concordance between endoscopic and histological atrophy [39]. This leaves histological evaluation of chronic gastritis with mild atrophic changes as the best method, although biopsy procedures tend to be unpleasant and unlikely to be undertaken without good reason. Autopsy samples may therefore provide relevant rates of prevalence. To the best of our knowledge, this is the first observational gastritis study using postmortem samples to examine the prevalence of histological chronic gastritis with or without atrophy in a cohort most closely representing the general population, including potentially asymptomatic cases. We found the prevalence of chronic non-atrophic or atrophic gastritis to be 40.0% in the whole series, which is higher than expected based on previous studies.

The limitations of this study include the fact that it is male dominated since prospective medicolegal autopsy series on victims of sudden out-of-hospital death mainly comprises of men. Males tend to have more dangerous habits including excessive use of alcohol and thus have a greater risk of dying in accidents and of non-natural causes. Men are also more likely to suffer an unexpected sudden cardiac death. Despite these biases however, a medicolegal autopsy series comprising unexpected out-of-hospital deaths is considered to be the best available series to represent the general population [40]. Because of the unexpected nature of the death, we have limited or no information on their smoking habits, medications and alcohol use. Additionally, we had no possibility to measure the presence of anaemia or vitamin B12 and folate levels in our autopsy cases. We do not believe that postmortem changes of the ventricular mucosa have led to an overdiagnosis of atrophic gastritis. Autolytic changes of the glands did not prevent the scoring of the samples because this was based on the presence or absence of inflammatory cells and lymphoid follicles and in the atrophic stage on the loss of appropriate glands and their replacement by fibrous tissue or intestinal-type glands (metaplasia).

Based on the present results, the possibility of insidious atrophic gastritis should be considered more often when examining older patients with heartburn or regurgitation as well as patients with microcytic or megaloblastic anaemia, pernicious anaemia or neurologic symptoms [13, 30]. Further studies are needed to confirm our findings on the common occurrence of asymptomatic atrophic gastritis in older people and its potential contribution to diseases related to dietary deficiencies that manifest as low levels of vitamin B12, folate and iron.

## Research Data Transparency and Availability

Data used for this analysis are available upon reasonable request with valid data transfer agreements. The data cannot be made publicly available due to ethical restrictions.

## References

[1] Kusters JG, van Vliet AHM, Kuipers EJ. Pathogenesis of *Helicobacter pylori* infection. Clin Microbiol Rev. 2006;19:449–90. 10.1128/CMR.00054-05. 16847081 PMC1539101

[2] Sipponen P, Maaroos HI. Chronic gastritis. Scand J Gastroenterol. 2015;50:657–67. 10.3109/00365521.2015.1019918. 25901896 PMC4673514

[3] Sipponen P, Kekki M, Haapakoski J et al. Gastric cancer risk in chronic atrophic gastritis: statistical calculations of cross-sectional data. Int J Cancer. 1985;35:173–7. 10.1002/ijc.2910350206. 3871738

[4] Correa P . Human gastric carcinogenesis: a multistep and multifactorial process–first American Cancer Society award lecture on cancer epidemiology and prevention. Cancer Res. 1992;52:6735–40. 1458460

[5] Uemura N, Okamoto S, Yamamoto S et al. *Helicobacter pylori* infection and the development of gastric cancer. N Engl J Med. 2001;345:784–9. 10.1056/NEJMoa001999. 11556297

[6] Nilsson-Ehle H, Landahl S, Lindstedt G et al. Low serum cobalamin levels in a population study of 70- and 75-year-old subjects. Digest Dis Sci. 1989;34:716–23. 10.1007/BF01540343. 2714146

[7] Lachner C, Steinle NI, Regenold WT. The neuropsychiatry of vitamin B12 deficiency in elderly patients. JNP. 2012;24:5–15. 10.1176/appi.neuropsych.11020052. 22450609

[8] Russell RM, Krasinski SD, Samloff IM et al. Folic acid malabsorption in atrophic gastritis: possible compensation by bacterial folate synthesis. Gastroenterology. 1986;91:1476–82. 10.1016/0016-5085(86)90204-0. 3770372

[9] Wang HX, Wahlin Å, Basun H et al. Vitamin B _12_ and folate in relation to the development of Alzheimer’s disease. Neurology. 2001;56:1188–94. 10.1212/WNL.56.9.1188. 11342684

[10] Sipponen P, Laxén F, Huotari K et al. Prevalence of low vitamin B12 and high homocysteine in serum in an elderly male population: association with atrophic gastritis and *Helicobacter pylori* infection. Scand J Gastroenterol. 2003;38:1209–16. 10.1080/00365520310007224. 14750639

[11] Ford ES, Smith SJ, Stroup DF et al. Homocyst(e)ine and cardiovascular disease: a systematic review of the evidence with special emphasis on case-control studies and nested case-control studies. Int J Epidemiol. 2002;31:59–70. 10.1093/ije/31.1.59. 11914295

[12] Rugge M, Correa P, Dixon MF et al. Gastric mucosal atrophy: Interobserver consistency using new criteria for classification and grading. Aliment Pharmacol Ther. 2002;16:1249–59. 10.1046/j.1365-2036.2002.01301.x. 12144574

[13] Shah SC, Piazuelo MB, Kuipers EJ et al. AGA clinical practice update on the diagnosis and management of atrophic gastritis: expert review. Gastroenterology. 2021;161:1325–1332.e7. 10.1053/j.gastro.2021.06.078. 34454714 PMC8740554

[14] Banks M, Graham D, Jansen M et al. British Society of Gastroenterology guidelines on the diagnosis and management of patients at risk of gastric adenocarcinoma. Gut. 2019;68:1545–75. 10.1136/gutjnl-2018-318126. 31278206 PMC6709778

[15] Neumann WL, Coss E, Rugge M et al. Autoimmune atrophic gastritis—pathogenesis, pathology and management. Nat Rev Gastroenterol Hepatol. 2013;10:529–41. 10.1038/nrgastro.2013.101. 23774773

[16] Presotto F, Sabini B, Cecchetto A et al. *Helicobacter pylori* infection and gastric autoimmune diseases: is there a link? Helicobacter. 2003;8:578–84. 10.1111/j.1523-5378.2003.00187.x. 14632671

[17] Toh BH, Chan J, Kyaw T et al. Cutting edge issues in autoimmune gastritis. Clin Rev Allergy Immunol. 2012;42:269–78. 10.1007/s12016-010-8218-y. 21174235

[18] Malaty HM, El-Kasabany A, Graham DY et al. Age at acquisition of *Helicobacter pylori* infection: a follow-up study from infancy to adulthood. Lancet. 2002;359:931–5. 10.1016/S0140-6736(02)08025-X. 11918912

[19] Fox JG, Wang TC. Inflammation, atrophy, and gastric cancer. J Clin Invest. 2007;117:60–9. 10.1172/JCI30111. 17200707 PMC1716216

[20] Rautelin H, Kosunen T. *Helicobacter pylori* infection in Finland. Ann Med. 2004;36:82–8. 10.1080/07853890310020293. 15119828

[21] Genta RM, Lash RH. *Helicobacter pylori*-negative gastritis: seek, yet ye shall not always find. Am J Surg Pathol. 2010;34:e25–34. 10.1097/PAS.0b013e3181e51067. 20631607

[22] Nordenstedt H, Graham DY, Kramer JR et al. *Helicobacter pylori*-negative gastritis: prevalence and risk factors. Am J Gastroenterol. 2013;108:65–71. 10.1038/ajg.2012.372. 23147524 PMC3984401

[23] Ferreira RM, Pereira-Marques J, Pinto-Ribeiro I et al. Gastric microbial community profiling reveals a dysbiotic cancer-associated microbiota. Gut. 2018;67:226–36. 10.1136/gutjnl-2017-314205. 29102920 PMC5868293

[24] Graham DY, Opekun AR, Osato MS et al. Challenge model for *Helicobacter pylori* infection in human volunteers. Gut. 2004;53:1235–43. 10.1136/gut.2003.037499. 15306577 PMC1774191

[25] Genta RM . *Helicobacter pylori*, inflammation, mucosal damage, and apoptosis: pathogenesis and definition of gastric atrophy. Gastroenterology. 1997;113:S51–5. 10.1016/S0016-5085(97)80012-1. 9394760

[26] Buti L, Spooner E, Van Der Veen AG et al. *Helicobacter pylori* cytotoxin-associated gene a (CagA) subverts the apoptosis-stimulating protein of p53 (ASPP2) tumor suppressor pathway of the host. Proc Natl Acad Sci U S A. 2011;108:9238–43. 10.1073/pnas.1106200108. 21562218 PMC3107298

[27] Owen DA . Gastritis and carditis. Mod Pathol. 2003;16:325–41. 10.1097/01.MP.0000062995.72390.14. 12692198

[28] Sipponen P, Kosunen TU, Valle J et al. *Helicobacter pylori* infection and chronic gastritis in gastric cancer. J Clin Pathol. 1992;45:319–23. 10.1136/jcp.45.4.319. 1577969 PMC495272

[29] Faraji EI, Frank BB. Multifocal atrophic gastritis and gastric carcinoma. Gastroenterol Clin North Am. 2002;31:499–516. 10.1016/S0889-8553(02)00008-0. 12134615

[30] Kryssia IRC, Marilisa F, Antonino N et al. Clinical manifestations of chronic atrophic gastritis. Acta Biomed. 2018;89:88–92. 30561424 10.23750/abm.v89i8-S.7921 PMC6502219

[31] Yin Y, Liang H, Wei N et al. Prevalence of chronic atrophic gastritis worldwide from 2010 to 2020: an updated systematic review and meta-analysis. Ann Palliat Med. 2022;11:3697–703. 10.21037/apm-21-1464. 36635994

[32] Song H, Held M, Sandin S et al. Increase in the prevalence of atrophic gastritis among adults age 35 to 44 years old in northern Sweden between 1990 and 2009. Clin Gastroenterol Hepatol. 2015;13:1592–1600.e1. 10.1016/j.cgh.2015.04.001. 25857683

[33] Green TJ, Venn BJ, Skeaff CM et al. Serum vitamin B12 concentrations and atrophic gastritis in older new Zealanders. Eur J Clin Nutr. 2005;59:205–10. 10.1038/sj.ejcn.1602059. 15483636

[34] Zagari RM, Rabitti S, Greenwood DC et al. Systematic review with meta-analysis: diagnostic performance of the combination of pepsinogen, gastrin-17 and anti-*Helicobacter pylori* antibodies serum assays for the diagnosis of atrophic gastritis. Aliment Pharmacol Ther. 2017;46:657–67. 10.1111/apt.14248. 28782119

[35] Bettington M, Brown I. Autoimmune gastritis: Novel clues to histological diagnosis. Pathology. 2013;45:145–9. 10.1097/PAT.0b013e32835cc22c. 23277173

[36] Axon ATR . Relationship between *Helicobacter pylori* gastritis, gastric cancer and gastric acid secretion. Adv Med Sci. 2007;52:55–60. 18217390

[37] Telaranta-Keerie A, Kara R, Paloheimo L et al. Prevalence of undiagnosed advanced atrophic corpus gastritis in Finland: an observational study among 4,256 volunteers without specific complaints. Scand J Gastroenterol. 2010;45:1036–41. 10.3109/00365521.2010.487918. 20446846

[38] Kosunen TU, Aromaa A, Knekt P et al. Helicobacter antibodies in 1973 and 1994 in the adult population of Vammala, Finland. Epidemiol Infect. 1997;119:29–34. 10.1017/S0950268897007565. 9287940 PMC2808819

[39] Carr NJ, Leadbetter H, Marriott A. Correlation between the endoscopic and histologic diagnosis of gastritis. Ann Diagn Pathol. 2012;16:13–5. 10.1016/j.anndiagpath.2011.08.002. 22079171

[40] Kok E, Haikonen S, Luoto T et al. Apolipoprotein E-dependent accumulation of Alzheimer disease-related lesions begins in middle age. Ann Neurol. 2009;65:650–7. 10.1002/ana.21696. 19557866

